# A computational method for prediction of matrix proteins in endogenous retroviruses

**DOI:** 10.1371/journal.pone.0176909

**Published:** 2017-05-04

**Authors:** Yucheng Ma, Ruiling Liu, Hongqiang Lv, Jiuqiang Han, Dexing Zhong, Xinman Zhang

**Affiliations:** School of Electronic and Information Engineering, Xi’an Jiaotong University, Xi’an, China; "INSERM", FRANCE

## Abstract

Human endogenous retroviruses (HERVs) encode active retroviral proteins, which may be involved in the progression of cancer and other diseases. Matrix protein (MA), in group-specific antigen genes (*gag*) of retroviruses, is associated with the virus envelope glycoproteins in most mammalian retroviruses and may be involved in virus particle assembly, transport and budding. However, the amount of annotated MAs in ERVs is still at a low level so far. No computational method to predict the exact start and end coordinates of MAs in gags has been proposed yet. In this paper, a computational method to identify MAs in ERVs is proposed. A divide and conquer technique was designed and applied to the conventional prediction model to acquire better results when dealing with gene sequences with various lengths. Initiation sites and termination sites were predicted separately and then combined according to their intervals. Three different algorithms were applied and compared: weighted support vector machine (WSVM), weighted extreme learning machine (WELM) and random forest (RF). *G* − *mean* (geometric mean of sensitivity and specificity) values of initiation sites and termination sites under 5-fold cross validation generated by random forest models are 0.9869 and 0.9755 respectively, highest among the algorithms applied. Our prediction models combine RF & WSVM algorithms to achieve the best prediction results. 98.4% of all the collected ERV sequences with complete MAs (125 in total) could be predicted exactly correct by the models. 94,671 HERV sequences from 118 families were scanned by the model, 104 new putative MAs were predicted in human chromosomes. Distributions of the putative MAs and optimizations of model parameters were also analyzed. The usage of our predicting method was also expanded to other retroviruses and satisfying results were acquired.

## Introduction

Human endogenous retroviruses (HERVs) are remnants of ancient retroviral infections. HERVs and their related genetic elements make up 504 distinct families and compose ~8% of human genome [1]. Typical full-length HERVs are about 7-11kb in size and consist mainly of the coding regions for *gag*, *pro*, *pol*, and *env* genes, flanked on both 5’- and 3’- ends by long terminal repeats (LTR). Most HERVs in human genome have incomplete structures [2], which contain multiple stop codons, insertions, deletions and frame shift mutations [3,4]. HERVs encode active retroviral proteins, which may exert important physiological functions in the body, but may also be involved in the progression of cancer and numerous human autoimmune, neurological and infectious diseases. In addition, HERVs regulate expression of the neighboring host genes and modify the genomic regulatory landscape [5].

The shortage of organs for transplantation is a major barrier to the treatment of organ failure. While porcine organs are considered promising, their use has been checked by concerns about transmission of porcine endogenous retroviruses (PERVs) to humans. The risk of infections of human recipients after xenotransplantations is now mainly represented by PERVs as these particles are part of the porcine genome. It was found that PERV infection of the HEK-293 cell line alters expression of HERV sequences [6]. However, this risk isn’t impossible to overcome, considering that the possibility that PERVs can be inactivated for clinical application to porcine-to-human xenotransplantation has been demonstrated in a recent research [7]. The close relationship between HERVs and PERVs reminds us of the importance of bioinformatics research to be carried out combining different mammal ERVs.

Group-specific antigen (*gag*) is the genetic material that codes for the core structural proteins of a retrovirus. *Gag* is one of the three "main" genes found in all retroviruses (along with env and pol). *Gag*s have close relationship to many serious diseases such as AIDS and cancer. A previous research revealed that human endogenous retrovirus K *gag* coassembles with HIV-1 *gag* and reduces the release efficiency and infectivity of HIV-1 [8]. It was found 2 years ago in another research that prostate cancer progression correlates with increased humoral immune response to a human endogenous retrovirus *gag* protein [9]. However, the amount of *gag* in HERVs found by experimental methods is still at a low level. The lack of annotated *gag*s in HERVs is a barrier that has to be removed for the convenience of subsequent structure analysis and function study on *gag*s in HERVs. A computational method to predict *gag*s from HERVs has been brought up [10], but no computational method to predict exact start and end coordinates of interior genes of *gag*s which encode functional proteins has been proposed yet. RetroTector is a platform independent program package which could detect candidate long terminal repeats (LTR) in retroviruses as well as chains of conserved retroviral motifs (including motifs of MAs) fulfilling distances constraints [11]. However, RetroTector is based on sequence alignment, thus only motifs instead of the exact start and end coordinates of MAs could be predicted. And these conserved retroviral motifs are eventually combined and used as basis of the detection of retroviruses.

*Gag* contains around 1500 nucleotides, and encodes three separate proteins which form the building blocks of the viral core. The three proteins are:

1. Matrix protein, MA
2. Capsid protein, CA
3. Nucleocapsid protein, NC

Matrix protein (MA) is associated with the virus envelope glycoproteins in most mammalian retroviruses and may be involved in virus particle assembly, transport and budding. Membrane binding in HIV-1 replication process is mediated by the MA, a 132-residue polypeptide containing an N-terminal myristyl group that can adopt sequestered and exposed conformations[12]. Single amino acid changes in the HIV-1 matrix protein block virus particle production[13]. The length of a MA found in endogenous retroviruses varies from 88aa to 127aa according to records in National Center for Biotechnology Information (NCBI). Computational method to predict interior genes of *gag*s which encodes functional proteins would benefit subsequent structure analysis and function study on *gag*s, but we have to overcome the difficulty of the shortage of annotated *gag* sequences in HERVs first.

Considering the importance of the relationship between *gag*s in HERVs and ERVs from other mammals, such as PERVs, *gag*s from different mammal ERVs could be combined to build up models for their interior gene prediction (i.e. MA).

In this paper, a computational model to identify MAs in ERVs was proposed. All ten parameters of divide physicochemical property scores (DPPS) [14] along with position weight matrix (PWM) were utilized to generate the feature space for MA prediction. An unconventional “divide and conquer” (D&C) technique was applied to acquire high prediction accuracy when dealing with sequences that are poorly conserved in their lengths (unlike the traditional D&C technique in computer science, D&C technique applied here is not intended to reduce the computational complexity of the algorithm, but to make conventional gene prediction algorithms designed for fix-length gene prediction also capable of predicting sequences with various lengths). Initiation sites and termination sites were predicted separately and then combined according to their intervals. Massive DNA sequences related with coding regions from 118 HERV families were scanned with the prediction model, which has high prediction accuracy under 5-fold cross validation test.

## Materials and methods

### Datasets

All available amino acid sequences of ERVs from various organisms were collected from NCBI at http://www.ncbi.nlm.nih.gov [15–68]. Among them, all 129 sequences of ERVs with MAs annotated in their *gag*s were used to build up the prediction model (please refer to S1 File for details). One hundred and twenty five of them are with both initiation sites and termination sites and the other four are with initiation sites only.

### “Divide and conquer”

In computer science, divide and conquer is an algorithm design paradigm based on multi-branched recursion. A divide and conquer algorithm works by recursively breaking down a problem into two or more sub-problems of the same or related type, until these become simple enough to be solved directly. The solutions to the sub-problems are then combined to give a solution to the original problem.

Traditional gene prediction methods would lose their accuracy or even feasibility when dealing with gene sequences with large length variations (because the dimension of the feature space couldn’t be constant), even though they could bring out ideal prediction results when dealing with fixed length gene sequences.

The idea of D&C inspired us to solve such problem. Unlike conventional D&C, the recursion level in our problem is only 2 because our main purpose is dividing the original problem into two simpler sub-problems instead of reducing the time complexity of it. Our prediction method focuses more on the boundaries of the genes instead of the interior areas, because the former has more to do with gene prediction. We broke down the problem into two simpler sub-problems (fixed length gene prediction) and then combined the solutions of them to generate prediction results of the original gene prediction problem. The two sub-problems are initiation site and termination site prediction, which could be done well with traditional gene prediction method because we could consider them as fixed length gene prediction problems. We just need to predict the fixed length flanking residues of the initiation site and termination site to deduce the precise locations of them. Then we just need to find a reasonable combination of the predicted initiation sites and termination sites to generate gene prediction results with high accuracy. We predicted the initiation site and termination site separately, and then regarded the sequence between them as a candidate MA sequence only when it has an appropriate length. The advantage of this divide and conquer technique is that feasibility and high accuracy could be obtained at the same time.

### Sample preparation

Training samples are prepared from the amino acid sequences with MA annotations. Positive training samples for initiation sites consisted of *s*-aa-long subsequences starting from the initiation sites. The best prediction result was obtained when *s* was set to be 15 (please refer to Discussion part for more details). Likewise, positive training samples for termination sites consisted of 15-aa-long subsequences ending at the termination sites. Negative training samples consisted of 15-aa-long subsequences from regions either without MAs or overlapping with MAs but not sharing the same initiation or termination sites with them. To overcome the difficulty of the lack of positive training samples, we generated negative training samples with a size 5 times as large as the positive sample size and took the imbalanced data problem into our consideration in the modelling process. Thus the training sets for initiation site prediction model and termination site prediction model were built separately.

### Feature selection

Combining position characteristics of sequences and physicochemical properties, a hybrid feature space construction approach was proposed.

Position weight matrix (PWM) [69] was applied to extract the position characteristic of sequences. A PWM has one row for each symbol of the alphabet: 20 rows for 20 kinds of amino acids in this case. It also has one column for each position of the 15-aa-long pattern. So a 20×15 matrix was built to represent the different frequencies of 20 kinds of amino acid appearing on various positions of the 15-aa-long motifs in this case. To construct a PWM, a basic position frequency matrix (PFM) is created by counting the occurrences of each nucleotide at each position at first. From the PFM, a position probability matrix (PPM) can be created by dividing that former nucleotide count at each position by the number of sequences, thereby normalising the values. Formally, given a set *X* of *N* aligned sequences of length *l*, the elements of the PPM *M*^*PPM*^ are calculated:

$$M_{k,j}^{PPM}=\frac{1}{N}\sum_{i=1}^{N}I(X_{i,j}=k)$$ (1)

Where *i* ∈(1,…,*N*), *j* ∈(1,…,*l*), *k* is the set of symbols in the alphabet and *I*(*a* = *k*) is an indicator function where *I*(*a* = *k*) is 1 if *a* = *k* and 0 otherwise.

Most often the elements in PWMs are calculated as log likelihoods. That is, the elements of the PWM are transformed using a background model *b* so that:

$$M_{k,j}^{PWM}=\text{log}(M_{k,j}^{PPM}/b_{k})$$ (2)

The above equation describes how an element in the PWM *M*^*PWM*^ is calculated. The simplest background model assumes that each letter appears equally frequently in the dataset. That is, the value of *b*_*k*_ = 1 / |*k*| for all symbols in the alphabet (|*k*| = 20 for amino acids, so *b*_*k*_ = 0.05).

After generating the PWM matrix with positive sequences (please refer to S5 File for details of PWM matrix of MA initiation sites and S6 File for details of PWM matrix of MA termination sites), a mapping method is used to extract the position characteristic of any 15-aa-long sequence *V*. Assign each amino acid of *V* with its corresponding value in the matrix according to its position. Then a 15-dimension-vector *V*^*Pos*^ to represent the position characteristic of the original 15-aa-long sequence could be generated.

$$V_{j}^{Pos}=M_{k,j}^{PWM}$$ (3)

Where *j* ∈ (1,…,*l*), *k* = *V*_*j*_, *l* = 15.

All ten parameters of the divided physicochemical property scores (DPPS) [14] were selected to extract the physicochemical properties of sequences. The parameters consist of 4 electronic properties, 2 steric properties, 2 hydrophobic properties and 2 hydrogen bond properties. Similarly, when dealing with a 15-aa-long sequence, the sequence was mapped into a 10×15 matrix to represent its physicochemical properties.

Combining the above two kinds of information, (1+10)×15 = 165 features in total were extracted from each 15-aa-long sequence for prediction. To get a persuasive performance comparison of different prediction models, we ran the following binary classifiers on the same 165-dimensional feature space.

### Binary classifiers

Three binary classifiers based on different principles were applied to predict the initiation sites and termination sites of the MA sequences:

#### WSVM classifier

The support vector machine (SVM) is a supervised machine learning algorithm based on the statistical learning theory [70]. The basic thought of SVM is to map the original data into a high dimensional feature space through a nonlinear mapping function and then construct a hyper plane as a discriminative surface between the positive and negative data [71]. Weighted SVM (WSVM) is able to deal with data with imbalanced class distribution while maintaining a good performance. In this paper, WSVM was employed to solve both the initiation site prediction and the termination site prediction, which is available at http://www.csie.ntu.edu.tw/~cjlin/libsvm/.

#### WELM classifier

Extreme learning machine (ELM) is a kind of artificial neural network and works for the “generalized” single-hidden-layer feed forward networks (SLFNs), the hidden layer (or called feature mapping) in ELM need not to be tuned. Compared with traditional computational intelligence techniques, ELM provides better generalization performance at a much faster learning speed. It has milder optimization constraints and with least human intervention [72]. Weighted ELM (WELM) also works well with data with imbalanced class distribution, and it is available at http://www.ntu.edu.sg/home/egbhuang/. In the paper, WELM was also used to solve the classification problem of unbalanced training samples of MA.

#### RF classifier

Random forest (RF) is an ensemble learning method for classification, regression and other tasks, that operate by constructing a multitude of decision trees at training time and outputting the class that is the mode) of the classes (classification) or mean prediction (regression) of the individual trees[73]. Random forest correct for decision trees' habit of overfitting to their training set. Random forest algorithm is also employed to solve the MA prediction problem, and it is available at https://cran.r-project.org/web/packages/randomForest/.

### Boundary combination

When the putative MA initiation sites and termination sites were predicted, a proper combination method should be proposed to get the prediction of the entire MA sequences. As we may acquire more than one putative initiation sites and more than one putative termination sites (making up even more putative boundary pairs) in one unannotated *gag* sequence, a method that could abandon the redundant false putative results and leave only one putative boundary pair as the final prediction results is required. Such requirement could be accomplished through the following 2 steps:

1. Choose the putative initiation sites and termination sites predicted by the RF models (please refer to S7 File for details of RF model for MA initiation sites and S8 File for details of RF model for MA termination sites) that possess distances within the range of the lengths of MA sequences (88 to 127 aa) as candidate boundary pairs.
2. Leave the candidate boundary pair that is predicted to be possible boundary pairs by WSVM models (please refer to S9 File for details of WSVM model for MA initiation sites and S10 File for details of WSVM model for MA termination sites) as well and also has the highest production value of its initiation site decision value and termination site decision value generated from the WSVM models as the final MA prediction result of the unannotated gag sequence. (A decision value is an important basis for the prediction result generated by a WSVM model. It is generated according to the degree of similarity between the predicted sample and training samples. It ranges from 0 to 1. The larger the decision value, the more likely the prediction result is positive, vise versa.)

Advantage of this technique:

1. Provides a way to rule out redundant MA boundary predictions and leave the most probable boundary pair as the final prediction result.
2. The final results have the advantages of both the RF models and WSVM models. They possess high sensitivity value provided by the RF models and high specificity value brought by the WSVM models (please refer to Results part for more details). By using the prediction results of RF models as candidate boundary pairs, we could reduce the omission rate of positive boundary pair. And by applying the WSVM model, false positive boundary pairs were ruled out as much as we could.

### Performance assessment

N-fold cross-validation and Jack-knife test are usually used to illuminate the performance of a prediction model. Since 5-fold and 10-fold cross-validation were found to work better than Jack- knife test [74], 5-fold and 10-fold cross-validation were employed to assess the performance of the models in this paper.

True positive (TP) and false negative (FN) are the number of positive samples that are predicted to be positive and negative respectively. Analogously, true negative (TN) and false positive (FP) are used to denote the number of negative samples that are predicted to be negative and positive respectively.

Sensitivity *S*_*n*_ (also called the true positive rate) measures the proportion of positive samples that are correctly identified as such. Specificity *S*_*p*_ (also called the true negative rate) measures the proportion of negative samples that are correctly identified as such.

Overall accuracy *ACC* denotes the proportion of the testing samples correctly predicted. Usually *ACC* is used to measure the effectiveness of a classifier. Unfortunately, in presence of imbalanced data, this metric may fail to provide adequate information about the performance of the classifier. For instance, when given a binary classification problem consisting of 1 percent positive sample and 99 percent negative class, any dumb classifier would easily achieve 99 percent accuracy by classifying all the samples as negative.

Matthew’s correlation coefficient *MCC* is also used in machine learning as a measure of the quality of binary classifications and it’s generally regarded as a balanced measure which can be used even if the classes are of very different sizes.

*G* − *mean* is an evaluation metric adopted in this paper to give more insight into the accuracy obtained within each class. As the geometric mean of the prediction accuracy of the positive samples and the prediction accuracy of the negative samples, *G* − *mean* could provide reasonable evaluation for the performance of the prediction model when dealing with imbalanced data. As with the 1:99 example, *G* − *mean* could be as low as 0 when the classifier is dump and could only classify all the samples as negative.

In this paper, *G* − *mean* under 5-fold cross-validation was selected as the major performance evaluation measure of the models to provide basis for parameter selection of models. *S*_*n*_, *S*_*p*_, *ACC* and *MCC* were also calculated as a supplemental reference.

$$\left\{ \begin{array}{l} S_{n}=\frac{TP}{TP+FN}\\ S_{p}=\frac{TN}{TN+FP}\\ ACC=(TP+TN)/(TP+TN+FN+FP)\\ MCC=\frac{\left(TP\times TN-FP\times FN\right)}{\sqrt{\left(TP+FP)(TP+FN)(TN+FP)(TN+FN\right)}}\\ G-mean=\sqrt{S_{n}S_{p}}=\sqrt{\frac{TP}{TP+FN}\times \frac{TN}{TN+FP}} \end{array}\right.$$ (4)

### Putative MA detection

Once the RF models with high accuracy for initiation and termination site prediction were trained, putative MA sequences could be obtained by applying sliding window technique. When an unannotated sequence was analyzed, a 15-aa-long sliding window was used to “observe” the sequence. As with the initiation site prediction, the prediction result of the 15-aa-long subsequence in the window could be acquired when the subsequence was put into the RF model which was previously trained to distinguish MA initiation sites. Then we can obtain the putative MA initiation site after sliding the window on the entire sequence. Similarly, we can obtain the putative MA terminal site on the same sequence as well. With the reasonable combination narrated in the ‘Boundary combination’ part, we could then finally decide whether the sequence between the putative MA initiation site and terminal site is a putative MA sequence or not.

## Results

### Performance of the method

#### Accuracy of the prediction of MA boundaries

The performance of the prediction models was tested by 5-fold cross-validation and shown in Table 1. From Table 1, we can find that RF models have the best prediction results. They have the highest *S*_*n*_, *ACC*, *MCC* and *G* − *mean* values for MA prediction of both initiation sites and termination sites (*G* − *mean* values of initiation sites and termination sites are 0.9869 and 0.9755 respectively), while WSVM models have the highest *S*_*p*_ values.

**Table 1:** Prediction performance of models applying different algorithms.

MA boundary type	Algorithm	Sn	Sp	ACC	MCC	G-mean
MA initiation sites	WSVM	0.9023	**1**	0.9837	0.9406	0.9497
	WELM	0.9605	0.9992	0.9928	0.9738	0.9795
	RF	**0.9767**	0.9974	**0.9939**	**0.9783**	**0.9869**
MA termination sites	WSVM	0.9152	**1**	0.9859	0.9484	0.9561
	WELM	0.9456	0.9922	0.9844	0.9439	0.9683
	RF	**0.9536**	0.9982	**0.9908**	**0.9667**	**0.9755**

#### Accuracy of the prediction of MA

All of the 125 ERV sequences collected with complete MAs were used to test the prediction performance of our prediction model (please refer to S2 File for more details). 123 of them were predicted completely correct. This means that 98.4% of the sequences could be predicted completely correct. The other 2 were predicted with only 2aa position deviations in their terminal sites. It is worth mentioning that all the initiation sites were predicted completely correct.

### Putative MA detection results

**Fig 1:**
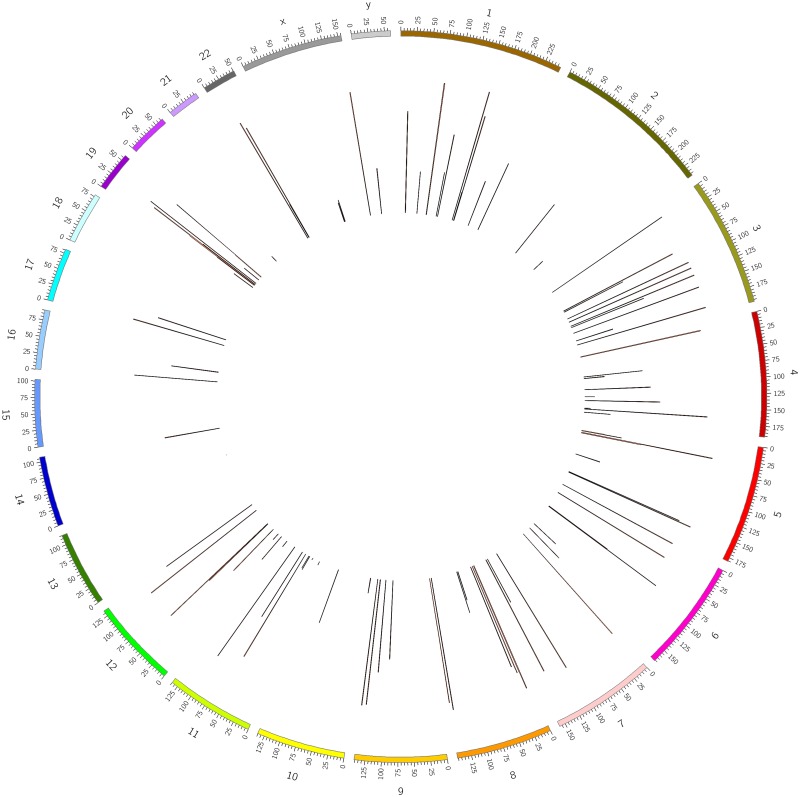
Exact locations of 104 new putative MAs of HERVs in the human chromosomes.

The proposed model was used to search for new putative MAs of 118 HERV families from sequences without MA annotations. A total of 94,671 DNA sequences (please refer to S3 File for details) corresponding to coding regions of HERVs from RepeatMasker have been scanned. 104 new putative MAs (please refer to S4 File for details) were predicted in coding region sequences. The exact locations of these new putative MAs of HERVs in the human chromosomes have been described with CIRCOS [75] software and shown in Fig 1.

The angles of the dark red lines represent the exact positions of new putative MAs of HERVs in the human chromosomes. The length of the line is proportional to the product of the decision values of the initiation site and termination site of the corresponding MA.

## Discussion

### Conservative property of MA boundaries

**Fig 2:**
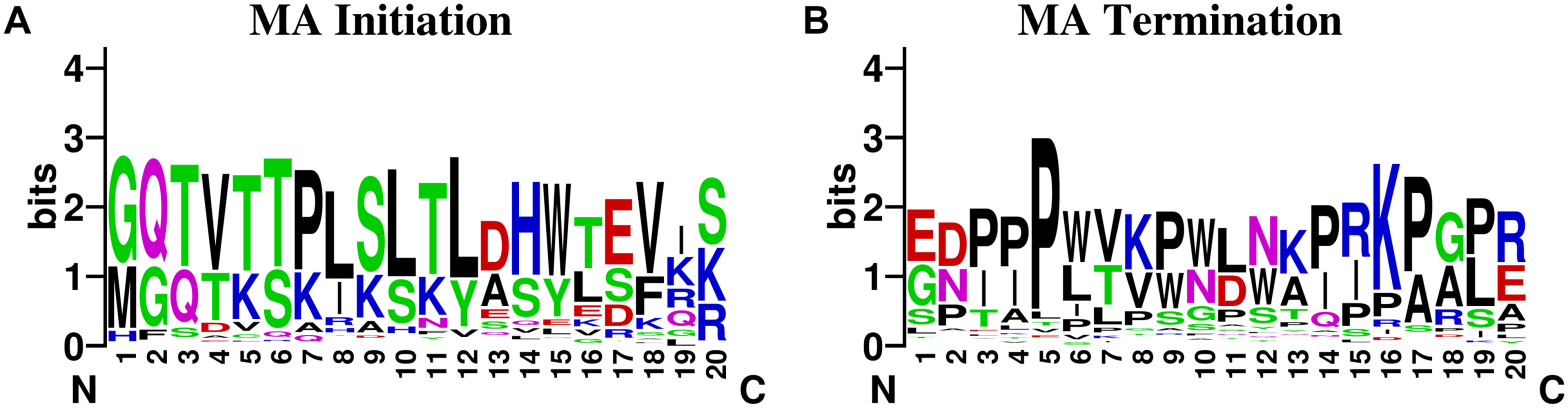
Motifs of residues adjacent to boundaries of MAs in ERV sequences. It shows motifs of surrounding residues of ERVs’ (A) MA initiation sites, (B) MA Termination sites.

Motifs of sequences adjacent to origins and terminals of MAs in ERVs were generated based on WebLogo version 2.8.2 (http://weblogo.berkeley.edu/logo.cgi) and shown in Fig 2. From the figure, we can defer that sequences on both ends of MAs in ERVs are fairly conservative. This explains why satisfying results could be obtained from models created to predict the origins and terminals of MAs in ERVs separately.

### Distribution of the putative MAs

**Fig 3:**
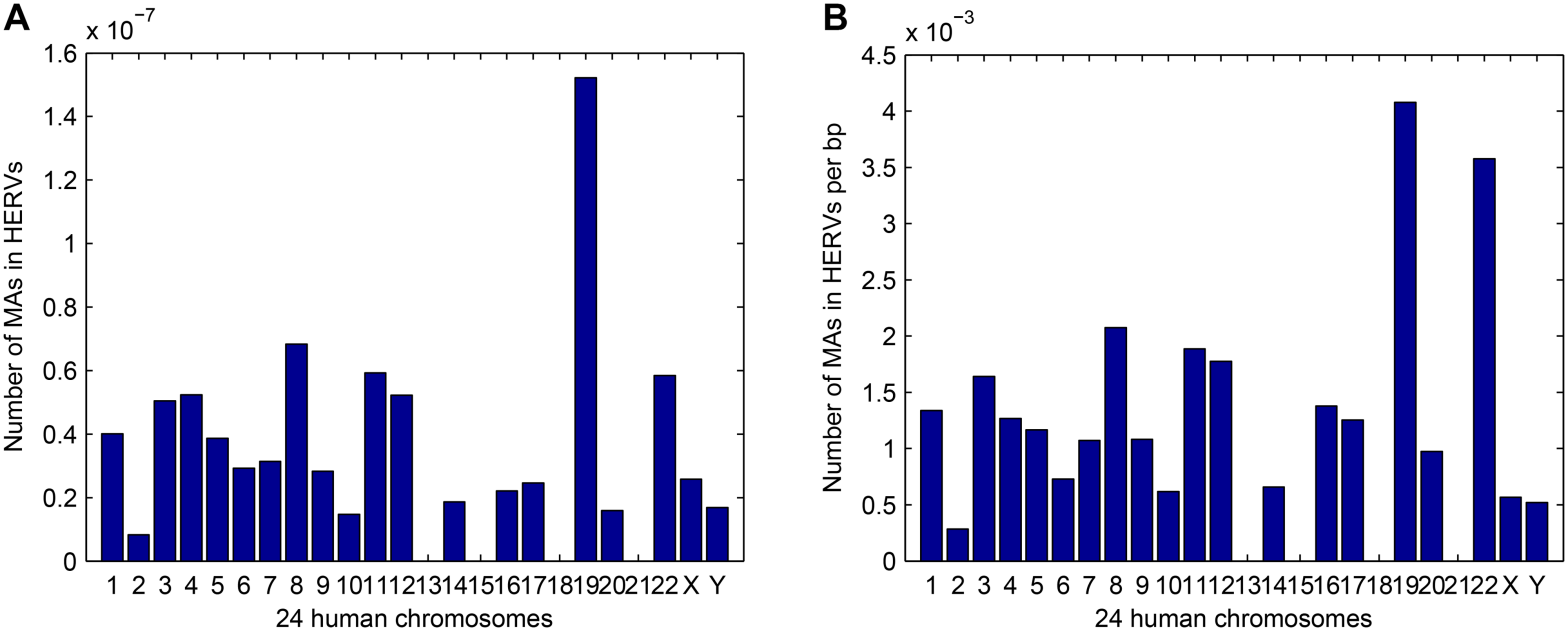
Distribution of MAs. (A) The number of MAs in HERVs of the 24 human chromosomes. (B)The number of MAs per bp in HERVs of the 24 human chromosomes.

The number of MAs in HERVs of the 24 human chromosomes and the number of MAs per bp in HERVs of the 24 human chromosomes were shown in Fig 3.

### Optimization of model parameters

Model parameters were optimized according to the prediction performance they eventually bring about. A parameter was settled when it could bring about the best prediction performance. To rule out random factors as much as possible, the whole prediction process was rerun for 10 times and the average of the model performance measurement values was calculated whenever a parameter value changes during the parameter optimization process. To choose the best values of parameters in the models, we adopted the method of cross validation based on grid search, avoiding the arbitrary and capricious behaviour.

The window length was selected according to its prediction performance. It was found that 15 is the best window length when the performance of the initiation site prediction model and the termination site prediction model are comprehensively considered.

The optimization details of model parameters in WSVM, WELM, RF models were shown in Table 2.

**Table 2:** Optimization details of parameters in WSVM, WELM, RF models.

MA boundary type	Algorithm	Model parameters	Step size in search	Value of optimized parameters
MA initiation sites	WSVM	c	0.0001	0.1895
		*g*	0.0001	0.0625
	WELM	Number of hidden neurons	50	2000
		*C*	50	9300
	RF	Number of trees	5	160
		*mtry*	5	80
MA termination sites	WSVM	c	0.0001	0.5743
		*g*	0.0001	0.1895
	WELM	Number of hidden neurons	50	1600
		*C*	50	5100
	RF	Number of trees	5	140
		*mtry*	5	50

### Predicting effectiveness on other retroviruses

Retroviruses from different genuses might have different gag structures [76]. To test the predicting effectiveness on other retroviruses, all available gag sequences with MA annotated in Retroviridae, including Alpharetrovirus, Betaretrovirus, Gammaretrovirus, Deltaretrovirus, Epsilonretrovirus, Lentivirus and Spumavirus were collected from NCBI and used as source of training and testing sets to examine the effectiveness of our method. (No MAs were found annotated in Epsilonretrovirus and Spumavirus. Please refer to S11 File for more details about annotated MAs in Retroviridaes.)

Prediction models based on similar strategy were built for MAs in retroviruses from various genuses. Their effectiveness was also tested (Prediction source code is available at SourceForge, with the download URL: https://sourceforge.net/projects/ma-detection/files/MA%20prediction.zip/download). The dataset summary, model parameters and prediction results were shown in Tables 3 & 4.

**Table 3:** Prediction performance of models applied to MA boundaries from different retrovirus genuses.

MA Boundary Type	Organism	Number of sequences	Algorithm	Sn	Sp	Acc	MCC	G-mean
MA Initiation Sites	Alpharetrovirus	141	WSVM	0.9931	1	0.9988	0.9958	0.9965
			RF	0.9931	1	0.9988	0.9958	0.9965
	Betaretrovirus	95	WSVM	0.9895	1	0.9928	0.9936	0.9947
			RF	0.9979	0.9994	0.9991	0.9969	0.9986
	Gammaretrovirus	482	WSVM	0.9726	0.998	0.9938	0.9775	0.9852
			RF	0.9807	0.9965	0.9938	0.9779	0.9885
	Deltaretrovirus	234	WSVM	0.9812	1	0.9969	0.9887	0.9905
			RF	0.9872	1	0.9979	0.9923	0.9936
	Lentivirus	17272	WSVM	0.9619	0.9987	0.9926	0.9732	0.9801
			RF	0.9746	0.9982	0.9942	0.9792	0.9863
MA Initiation Sites	Alpharetrovirus	140	WSVM	0.9857	1	0.9976	0.9914	0.9928
			RF	0.9907	1	0.9985	0.9944	0.9953
	Betaretrovirus	98	WSVM	0.99	1	0.9983	0.9939	0.9949
			RF	1	1	1	1	1
	Gammaretrovirus	347	WSVM	0.9447	0.9959	0.9873	0.954	0.9699
			RF	0.9516	0.9978	0.9901	0.9639	0.9743
	Deltaretrovirus	181	WSVM	0.9892	0.9945	0.9936	0.9773	0.9918
			RF	0.9891	0.9989	0.9972	0.9901	0.9939
	Lentivirus	18234	WSVM	0.9074	0.9998	0.9844	0.9433	0.9523
			RF	0.9625	0.9983	0.9923	0.9723	0.9802

**Table 4:** Prediction performance on sequences with intact MAs from different retrovirus genuses.

Organism	Intact Seq Amount	Init Acc Amount	Init Acc Rate	Term Acc Amount	Term Acc Rate	Boundaries Acc Amount	Boundaries Acc Rate
Alpharetrovirus	139	139	1	138	0.9928	138	0.9928
Betaretrovirus	95	95	1	95	1	95	1
Gammaretrovirus	341	336	0.9853	336	0.9853	332	0.9736
Deltaretrovirus	179	178	0.9944	171	0.9553	170	0.9497
Lentivirus	16292	15057	0.9242	15190	0.9324	14196	0.8713

From Tables 3 & 4, we can find that our prediction models could bring out favourable prediction results on sequences from various genuses of Retroviridae. Thus our predicting strategy (focusing on predicting MA start and end coordinates by combining RF and WSVM) is extensible to various genuses of Retroviridae.

Gammaretroviruses, such as murine leukemia viruses (MLVs), encode, in addition to the canonical gag, pol, and env proteins that will form progeny virus particles, a protein called “glycogag” (glycosylated Gag) [77]. All available glycosylated Gag sequences with MA annotated were downloaded from NCBI and scanned by our prediction model for Gammaretrovirus. All of their annotated boundaries were predicted totally correct. (Please refer to S12 File for more details about MA prediction in glycosylated Gags). It seems that the prediction of MAs in glycogag is not a special issue distinguished from normal Gags. This consist with the previous research that glycogag protein is identical in primary sequence to Gag except that it contains 88 additional residues at its N terminus [78].

### Limits of the model

MA prediction based on identifying boundaries of MAs has the advantage of high efficiency and accuracy. However, some ERVs may not have a typical MA, like HERVL. Our prediction focuses on prediction of MAs with typical structures, thus it is not suitable for predicting non-canonical MAs.

## Supporting information

S1 File ERV sequences with MA annotations collected for research in this paper. (XLS)

S2 File MA prediction results of all 125 ERV sequences collected with complete MAs in this paper. (XLS)

S3 File Details of 94,671 DNA sequences corresponding to coding regions of HERVs from RepeatMasker. (FSA)

S4 File Details of 104 putative MAs in HERVs. (XLS)

S5 File Details of PWM matrix of MA initiation sites. (MAT)

S6 File Details of PWM matrix of MA termination sites. (MAT)

S7 File Details of RF model for MA initiation sites. (MAT)

S8 File Details of RF model for MA termination sites. (MAT)

S9 File Details of WSVM model for MA initiation sites. (MAT)

S10 File Details of WSVM model for MA termination sites. (MAT)

S11 File Details of all collected Retroviridae sequences with MAs annotated. (XLSX)

S12 File Details about MA prediction in glycosylated Gags. (XLSX)
